# Developmental Differences in Morphological Predictors of Power, Change-of-Direction Speed, and Reactive Agility in Youth Male Basketball Players

**DOI:** 10.3390/jfmk11020244

**Published:** 2026-06-21

**Authors:** Sousana Symeonidou, Afroditi Lola, Georgia Stavropoulou, Anastasios Dalkiranis, Marios Bismpos, Eleni Bassa

**Affiliations:** 1 School of Physical Education and Sport Science, Aristotle University of Thessaloniki, 57001 Thessaloniki, Greece; 2 School of Philosophy and Education, Aristotle University of Thessaloniki, 54124 Thessaloniki, Greece; 3 School of Physical Education and Sport Science at Serres, Aristotle University of Thessaloniki, 62500 Serres, Greece

**Keywords:** body composition, fat-free mass, jump, motor performance, reactive agility, adolescence

## Abstract

**Background**: Morphological characteristics influence physical performance in youth basketball, but their effects may differ by developmental stage. This study compared the predictive role of morphological variables on motor performance between U13 and U15 male players. **Methods**: Male youth basketball players (*N* = 89) were assigned to U13 and U15 groups. Morphological variables included height, body mass, body fat percentage, and fat-free mass (FFM). Motor tests evaluated squat jump (SJ), countermovement jump (CMJ), 10 m and 20 m sprints, T-test, Y-test and Stop-and-Go change-of-direction speed and reactive agility (RA). Pearson correlations and multiple linear regressions assessed relationships and predictive effects. **Results**: In U13 players, several morphological variables correlated with performance: height and FFM were positively related to jumping and sprinting, while body fat was negatively associated with most measures (*p* < 0.05). Regression models explained substantial variance in sprint (ranging up to _Adj_R^2^ = 0.44) and jump performance (ranging up to _Adj_R^2^ = 0.32), though individual predictors were not always significant (*p* > 0.05). In U15 players, fewer associations emerged as body fat remained a significant negative predictor of jumping and agility, and greater body mass was associated with improved sprint performance (*p* < 0.05). No significant morphological predictors were found for RA in either group (*p* > 0.05). **Conclusions**: Morphological traits exert a stronger, multifactorial influence on performance in younger athletes, whereas body composition and particularly body fat are more influential in older adolescents. These results underscore the need to consider the developmental stage when assessing and training male youth basketball players.

## 1. Introduction

Basketball is a physiologically demanding, high-intensity intermittent sport characterized by repeated bouts of sprinting, jumping, accelerations, decelerations, and rapid changes of direction performed under time and spatial constraints [1,2]. Match-play analyses have shown that elite basketball players cover approximately 4,000–5,000 m during a competitive game, depending on playing level, position, and competition format, with guards generally covering greater distances than forwards and centers [3,4]. Players perform approximately 40–60 jumps, 50–105 high-intensity accelerations and decelerations, and 30–60 changes of direction, while completing 35–50 short-duration sprints, most commonly over distances of 5–15 m [3,5,6]. Furthermore, match activity profiles indicate that approximately 15–20% of total playing time is spent in high-intensity activities (sprinting, shuffling, jumping, and explosive directional changes), whereas the remaining 80–85% consists of low-intensity movements such as standing, walking, and jogging interspersed with brief recovery periods [3,7]. These repeated explosive efforts place substantial demands on both anaerobic power and neuromuscular efficiency. Beyond these physical demands, modern basketball performance is increasingly understood as a multidimensional construct, integrating physical, technical, tactical, and perceptual-cognitive components [8].

Speed, agility, and lower limb explosive power are fundamental motor abilities that significantly influence performance in team sports such as basketball [9]. These qualities are closely interrelated due to shared neuromuscular and biomechanical mechanisms, including stretch-shortening cycle (SSC) efficiency and rate of force development [10]. Therefore, strong associations have been reported among vertical jump performance, sprint speed, and change of direction (COD) ability in basketball players [11] because these abilities rely on similar neuromuscular characteristics, including explosive lower-limb power, rapid force production, and effective stretch-shortening cycle utilization [12]. Athletes capable of producing high levels of force in a short time are generally able to jump higher, sprint faster, and change direction more efficiently [12]. However, recent evidence suggests that these relationships can vary substantially across developmental stages, reflecting both the effects of sport-specific training and age-related changes in body size and composition [13].

Change-of-direction speed (CODS) and agility are related but distinct constructs. According to contemporary definitions, CODS refers to rapid pre-planned movements involving acceleration, deceleration, and directional changes without the requirement to respond to an external stimulus. In contrast, agility includes a perceptual and decision-making component and requires an appropriate movement response to an unpredictable stimulus. Previous research has demonstrated the validity of CODS assessments as measures of physical change-of-direction ability, while agility performance is additionally influenced by perceptual-cognitive processes [14,15]. Therefore, CODS and RA should be evaluated separately, particularly in youth athletes, where physical and cognitive determinants may develop at different rates [9,10,11,12]. Research further highlights that RA is less dependent on morphological characteristics and more strongly influenced by cognitive-perceptual abilities [16]. Despite extensive research in adult and elite populations, there remains a limited understanding of how these performance components interact with morphological characteristics in youth athletes, particularly during critical developmental windows.

Adolescence is a crucial period for athletic development, marked by rapid changes in body composition, height, and neuromuscular function that directly affect physical performance [17]. Substantial variability in performance can occur within the same chronological age group, which may bias talent identification and development processes [18]. Morphological characteristics, including height, body mass, body fat percentage, and fat-free mass, have been consistently identified as key contributors to performance in youth basketball [19]. Previous studies have reported that lower body fat percentage is associated with faster sprint times, greater jump performance, and superior change-of-direction ability in adolescent basketball players. Conversely, greater fat-free mass has been linked to enhanced force production, power output, and overall athletic performance. Furthermore, body composition has been identified as an important factor differentiating competitive levels and playing positions in youth basketball populations [18,19].

From a mechanistic perspective, body composition plays a central role in determining movement efficiency. Higher fat-free mass enhances force production and power output, while excess body fat imposes additional metabolic demands, negatively affecting sprinting, jumping, and agility performance in young athletes [20,21,22,23].

Significantly high body fat levels detrimentally affect explosive performance, as increased fat mass can increase inertia, necessitating greater force per kilogram of lean mass to alter movement velocity [14]. Additionally, structural characteristics such as height and limb length may influence leverage and movement mechanics, particularly during jumping and directional changes [24].

Although several studies have examined the associations between morphological characteristics and physical performance in youth basketball players, most have focused on simple correlational analyses or comparisons between competitive levels, playing positions, and age groups. Previous findings consistently indicate that greater height and fat-free mass, as well as lower body fat percentage, are associated with superior jumping, sprinting, and change-of-direction performance. However, the majority of these studies have investigated isolated performance outcomes and have not simultaneously evaluated the relative contribution of multiple morphological variables using multivariate predictive models. Moreover, research has predominantly focused on preplanned physical tasks, whereas the potential influence of morphological characteristics on RA remains largely unexplored. Thus, there is a need to evaluate young athletes using multiple agility tasks to differentiate between preplanned CODS and RA. This distinction is important because CODS predominantly reflects movement execution under anticipated demands, whereas RA additionally requires rapid perception, decision-making, and stimulus-driven response selection. Understanding how these relationships evolve during adolescence is essential for designing age-appropriate training programs and for avoiding premature talent-selection biases. In light of these considerations, this study aimed to systematically assess which morphological parameters can effectively predict physical fitness factors, such as jumping, speed, CODS, and RA, in young male basketball athletes providing a developmental perspective on the contribution of body composition and morphology to performance. Additionally, by exploring these associations, the study seeks to provide meaningful insights into optimizing training regimens for adolescent basketball players and ultimately supporting their progression from emerging talent to established senior competitors.

Based on previous evidence, it was hypothesized that morphological characteristics, particularly body fat percentage and fat-free mass, would significantly predict jumping, sprinting, and change-of-direction speed performance in youth basketball players. Furthermore, stronger associations were expected in the U13 group compared with the U15 group, reflecting the greater influence of morphological factors during earlier stages of development. In contrast, only limited associations were expected between morphological characteristics and RA performance, given the substantial contribution of perceptual-cognitive processes to this ability.

## 2. Materials and Methods

### 2.1. Sample

This study included 89 male basketball athletes aged 11–15 years, who competed in the local championship of Thessaloniki (EKASTH). The athletes attended at least 4 training sessions weekly and participated in one game during the weekend. All participants and their guardians were informed about the research purposes, the measurement protocol, and potential risks, and they signed a consent form. The study was conducted in accordance with the Declaration of Helsinki and approved by the Ethics Committee of the Department of Physical Education and Sports Science of Thessaloniki (Approval No.: 254/31 March 2025).

### 2.2. Experimental Design

All measurements were conducted in indoor basketball courts during the afternoon. Participants were familiarized with the testing procedures one week before the assessments. Body mass, height, and body fat percentage were recorded, and the following motor abilities were assessed: speed at 10 m and 20 m, lower limb power through Squat Jump (SJ) and Countermovement jump (CMJ), change-of-direction speed through the T-test, and RA through the Y-shaped agility test and the Stop and Go agility test. The measurements were conducted on two separate days, one week apart. On the first day, morphological characteristics were recorded, and the Y-shaped agility test, T-test, and sprints of 10 m and 20 m were performed. The SJ, CMJ and the Stop and Go agility test were conducted on the second day. The order of tests within each session was randomized to reduce potential order effects. Participants performed three trials for each test, with the best performance recorded for analysis. Adequate passive recovery periods (2–3 min between trials and 3–5 min between different tests) were provided to minimize fatigue influence. Before starting the tests, athletes performed a 15 min warm-up that included jogging, dynamic stretching, lateral displacement, and acceleration–deceleration. All athletes were familiarized with the tests before the measurements.

#### 2.2.1. Anthropometry

Body mass was measured to the nearest 0.1 kg, and height to the nearest 0.1 cm, with participants wearing light clothing and barefoot (Seca 220e, Hamburg, Germany). BMI was calculated using the formula kg/m^2^. Body fat percentage was estimated by recording the thickness of four skinfolds (biceps, triceps, suprailiac, subscapular) on the right side of the body using a specialized instrument ((Model 01127A, Lafayette Instrument Company, Lafayette, IN, USA). Body density was calculated using the equations of [25] for males under 16 years, and the Siri equation [26] was used for estimating body fat percentage.

#### 2.2.2. Biological Maturation

Biological maturation was estimated from the age at peak height velocity (APHV), calculated by subtracting the maturity offset (MO) from the chronological age on the test day. The maturity offset (YAPHV) was calculated using the equation −7.999994 + (0.0036124 × (age × height)) of [27] for boys.

#### 2.2.3. Speed Testing

Speed assessment was conducted through a maximum 20 m sprint, with time recorded using three pairs of photocells placed at the start, 10 m, and 20 m (Microgate, Bolzano, Italy). Participants started from a standing position with their dominant foot 0.3 m behind the first photocell gate and sprinted maximally through the third gate. The photocells were placed approximately 0.6 m above the ground, with a 1.5 m distance between them. Each participant performed two attempts with a 3 min rest between them, with verbal encouragement provided throughout. The previous test–retest reliability score for the sprint test in youth was 0.92 [28]. The intraclass correlation coefficients (ICC1) demonstrated good to excellent test–retest reliability across Sprint 10 m (ICC = 0.883; 95% CI: 0.828–0.922), Sprint 20 m (ICC = 0.943; 95% CI: 0.914–0.962).

#### 2.2.4. Lower Limb Power

Two vertical jumps, SJ and CMJ, were performed to assess lower limb power. In SJ, participants started from a semi-squat position (90° knee angle) and executed a maximal vertical jump without further bending the knees. In CMJ, participants performed a quick preparatory movement, bending the hips and knees at their preferred knee angle, followed by an explosive jump. Hands were placed on the hips for both jumps. Each participant performed two attempts per jump. Jump height was recorded using the Chronojump electronic leap mat (Chronojump, Boscosystem, Barcelona, Spain). Previous reliability score for jumping assessment using Chronojump was 0.99 [29]. The intraclass correlation coefficients (ICC1) demonstrated good test–retest reliability across SJ (ICC = 0.839; 95% CI: 0.765–0.891) and CMJ (ICC = 0.821; 95% CI: 0.740–0.879).

#### 2.2.5. Change-of-Direction Speed Test (T-Test)

The T-test was used to evaluate change-of-direction speed (CODS). Participants started behind point A, sprinted 9.14 m to point B, touched a 30 cm cone with their right hand, shuffled left 4.57 m to point C, touched the cone with their left hand, shuffled 9.14 m to point D, touched the cone with their right hand, shuffled left 4.57 m to point B, and finally sprinted backward to point A. Each participant performed two attempts with a 3 min rest between them. Time was recorded using a photocell gate at point A (Microgate, Bolzano, Italy). Previous reliability score of the T-test assessment in youth was 0.96 [28]. The intraclass correlation coefficients (ICC1) demonstrated excellent test–retest reliability (ICC = 0.915; 95% CI: 0.874–0.943).

#### 2.2.6. Y-Shaped Agility Test

The Y-Shaped Agility Test is a reliable assessment of the ability to change direction in preplanned and reactive conditions [30]. The total distance was 10 m, with participants sprinting 5 m straight to a trigger gate and then changing direction to the right or left at a 45° angle for another 5 m. In preplanned conditions, participants knew the direction in advance. Each performed three changes to the right and three to the left, with the best attempts used for analysis. Time was recorded using three photocell gates (Microgate, Bolzano, Italy).

For the reactive condition, participants reacted to visual stimuli from the FitLight Trainer system (Sports Corp., Bolton, ON, Canada). A LED at the 5 m mark deactivated upon passing, activating one of the two LEDs at the finish gates. Participants executed a split step at the trigger gate and then sprinted to the activated LED gate. Each performed six trials, three to each side, with the fastest trial for each direction used in the analysis. The reactive index (RI) was calculated as the difference between the best reactive and preplanned times. The previous reliability score for both Y-Shaped Agility Tests was 0.81–0.91 [31]. The intraclass correlation coefficients (ICC1) demonstrated good test–retest reliability across Y-test CODS right (ICC = 0.893; 95% CI: 0.841–0.928) and Y-test CODS left (ICC = 0.883; 95% CI: 0.827–0.921).

#### 2.2.7. Stop ‘n Go Agility Test

The Stop ‘n Go Agility Test consists of a preplanned and a reactive protocol [32]. In preplanned protocol, the athlete started from the start line when ready, triggering the photocell gate at 1.5 m. They ran to one of four LEDs, deactivated it by hand, and returned to the start line. Three scenarios (A-B-C, D-C-B, C-B-A) were executed, with the best time used for analysis.

In the reactive protocol, participants reacted to which LED activated and ran to deactivate it by hand. Three scenarios (B-D-B, A-B-D, D-A-C) were performed in a random order. Participants were divided into two groups, alternating protocols. The FitLight Trainer system (Sports Corp., Bolton, ON, Canada) provided visual stimuli. The best time for each scenario was used in the analysis. High reliability scores were previously reported (0.87–0.92) [33]. The intraclass correlation coefficients (ICC1) demonstrated good test–retest reliability across STOPGO CODS (ICC = 0.814; 95% CI: 0.730–0.874) and STOPGO RA (ICC = 0.730; 95% CI: 0.617–0.814).

### 2.3. Data Analysis

Data were analyzed by dividing participants into two age categories: U13 (*N* = 41, 46.1%) and U15 (*N* = 48, 53.9%), as age is an important factor affecting morphological characteristics and motor performance in young athletes. Descriptive statistics (mean, standard deviation, skewness, kurtosis, minimum, and maximum values) were calculated for all study variables. Normality tests indicated that assumptions were met (*p* > 0.05), while multicollinearity diagnostics showed acceptable variance inflation factor (VIF) values.

Pearson correlation analyses were conducted to examine linear relationships among variables. Multiple linear regression analyses were subsequently performed to investigate the extent to which morphological characteristics predicted performance and agility outcomes. Predictors were entered simultaneously using the enter method for theory-driven models. All predictor variables were included based on a priori theoretical and biomechanical considerations regarding their expected relationships with performance outcomes. Specifically, predictors were selected based on established physiological and biomechanical evidence indicating that height may influence jump performance through leverage and segment-length advantages, body fat percentage may impair sprint and agility performance due to increased inertial demands, and fat-free mass may positively contribute to force production and explosive performance. Adjusted R^2^ values were used to assess explained variance, whereas standardized beta coefficients (β), t-values, and *p*-values were examined to evaluate the direction, strength, and statistical significance of the predictors. Collinearity diagnostics indicated that all VIF and tolerance values were within acceptable limits, suggesting no multicollinearity issues. Statistical analyses were conducted using JASP (Version 0.96; JASP Team, Amsterdam, The Netherlands) via R.

## 3. Results

The initial stage of the data analysis involved descriptive analysis, which provided information on the mean, standard deviation, skewness, and kurtosis, maximum and minimum values for each variable under investigation, specifically for (a) descriptive morphological characteristics and (b) performance. Detailed information is shown in Table 1, Table 2, Table 3, and Table 4, respectively.

Pearson correlation analyses examined the relationships between morphological characteristics and motor performance variables (SJ, CMJ, 10 m sprint, 20 m sprint, and T-test) in the U13 and U15 groups. In the U13 group, height was positively associated with SJ and CMJ (r = 0.39, *p* < 0.05) and negatively associated with 10 m sprint (r = −0.47, *p* < 0.01), 20 m sprint (r = −0.48, *p* < 0.001), and T-test performance (r = −0.57, *p* < 0.001). Body mass was negatively related to sprint and change-of-direction speed performance (r = −0.33 to −0.45, *p* < 0.05). Body fat percentage was negatively correlated with SJ and CMJ (r = −0.47 to −0.49, *p* < 0.01) and positively associated with sprint performance (r = 0.35 to 0.46, *p* < 0.05), whereas fat-free mass showed the opposite pattern, being positively associated with jump performance (r = 0.32 to 0.36, *p* < 0.05) and negatively associated with sprint and change-of-direction speed measures (r = −0.48 to −0.55, *p* < 0.001). Body fat percentage was negatively correlated with SJ (r = −0.36, *p* < 0.05) and CMJ (r = −0.48, *p* < 0.01), while positively associated with T-test performance (r = 0.40, *p* < 0.01). No significant associations were observed for height or fat-free mass. This information is depicted in Table 5.

Pearson Correlations analysis was also used to investigate the correlations among morphological characteristics, change of direction speed (CODS) and RA in two different motor tests (Y-test, Stop and go test). Specifically, in the U13 group, body mass showed significant negative correlations with Y-TEST CODS Right (r = −0.34, *p* < 0.05), Y-TEST CODS Left (r = −0.31, *p* < 0.05), Y-TEST CODS Best (r = −0.37, *p* < 0.05), Y-TEST RA Left (r = −0.40, *p* < 0.01), and Y-TEST RA Best (r = −0.36, *p* < 0.05), while in the U15 group there were not statistically significant correlations. In the same manner, in U13 age group, height strongly associated with Y-TEST CODS Right (r = −0.46, *p* < 0.01), Y-TEST CODS Left (r = −0.36, *p* < 0.05), Y-TEST CODS Best (r = −0.47, *p* < 0.01), Y-TEST RA Left (r = −0.37, *p* < 0.05), and Y-TEST RA Best (r = −0.37, *p* < 0.05). On the other hand, in U15 there were not statistically significant correlations. Body fat percentage showed more pronounced adverse relationships in U15 age group than in U13. Specifically, in U13 body fat percentage correlated only with Y-TEST CODS Right (r = 0.36, *p* < 0.05), whereas in U15 correlations were observed with Y-TEST CODS Right (r = 0.39, *p* < 0.01), Y-TEST CODS Left (r = 0.29, *p* < 0.05), Y-TEST CODS Best (r = 0.29, *p* < 0.05), and Stop-Go Best CODS (r = 0.34, *p* < 0.05). Regarding FFM strong associations were observed with Y-TEST CODS Right (r = −0.46, *p* < 0.01), Y-TEST CODS Left (r = −0.40, *p* < 0.05), Y-TEST CODS Best (r = −0.47, *p* < 0.01), Y-TEST RA Left (r = −0.45, *p* < 0.01), and Y-TEST RA Best (r = −0.44, *p* < 0.01) in U13 group, whereas there were not statistically significant correlations in U15 group. For time-based performance variables (e.g., sprint, T-test, and agility assessments), negative correlation coefficients indicate better performance outcomes (shorter execution times), whereas positive correlations indicate poorer performance (longer execution times). This information is represented in Table 6.

The multiple linear regression analyses were used in order to investigate the influence of morphological characteristics on motor skills. Specifically, significant relationships were revealed between body mass, height, body fat, and fat-free mass (FFM) and performance in Squat Jump in U13. The overall model was statistically significant, F(5,35) = 5.09, *p* < 0.01, explaining 29% (_Adj_R^2^) of the variance in SJ. However, none of the individual predictors reached statistical significance. In U15, a multiple regression analysis was conducted to examine whether body mass, height, fat, and FFM predicted SJ performance. The overall regression model was statistically significant, F(4,43) = 4.16, *p* < 0.01, indicating that the predictors collectively explained a significant proportion of the variance in SJ performance. The model explained the 21.2% of the variance (_Adj_R^2^). Again, none of the individual predictors reached statistical significance. Morphological characteristics were also tested for their influence in CMJ. The best model for U13 was the one having as independent variables height and body fat, with *F*(2,38) = 10.21, *p* < 0.001, explaining 31.5% (_Adj_R^2^) of the variance. In this model, body fat was a significant negative predictor (β = −0.45, *t* = −3.43, *p* < 0.001), while height emerged as a significant positive predictor of CMJ performance (β = 0.33, *t* = 2.53, *p* < 0.05). For U15 group linear regression analysis showed that body fat significantly predicted CMJ performance, F(2,47) = 7.55, *p* < 0.001, explaining 21.8% of the variance. Body fat was a significant negative predictor of CMJ (β = −0.48, t = −3.70, *p* < 0.001), whereas height did not present any influence. This information is depicted in Table 7.

Regarding Sprint 10, the model with body mass, height, body fat, and fat-free mass (FFM) as independent variables predicted 10 m sprint performance in U13. The overall model was statistically significant, *F*(4,40) = 8.00, *p* < 0.001, explaining 41.2% (_Adj_R^2^) of the variance. However, only fat reached statistical significance at the 0.05 level (β = 1.52, *t* = 2.32, *p* < 0.05). For the U15 group, the regression model was also statistically significant, *F*(4,47) = 3.85, *p* < 0.01, explaining 19.5% (_Adj_R^2^) of the variance in 10 m sprint performance. Among the predictors, only FFM was a significant negative predictor (β = −3.57, *t* = −2.44, *p* < 0.05). For the U13 group, the regression model including FFM, Fat, Height, and body mass significantly predicted 20 m sprint performance, with _Adj_R^2^ = 43.8%, F(5,35) = 7.24, *p* < 0.001. Specifically, body mass was a significant negative predictor (β = −0.53, t = −4.24, *p* < 0.001) as it was associated with enhanced sprint performance, whereas fat was a significant positive predictor (β = 0.63, t = 5.05, *p* < 0.001). For the U15 group, the model was statistically significant, F(2,45) = 7.00, *p* < 0.01, indicating that the predictors significantly explained variance in sprint performance. The model yielded an adjusted R^2^ = 0.203 of the variance in Sprint 20. Regarding the individual predictors, body mass was a significant negative predictor of sprint performance (β = −0.66, t = −3.20, *p* < 0.01) and it was associated with enhanced sprint performance, and fat mass was a significant positive predictor (β = 0.76, t = 3.70, *p* < 0.001). Regarding T-test, for the U13 it was shown that the regression model was statistically significant, F(2,38) = 10.830, *p* < 0.001, indicating that the predictors significantly explained variance in T-test performance. The model produced an adjusted R = 0.330 showing that 33% of the variance in T-test performance was explained by the independent variables. Regarding the individual predictors, height was a significant negative predictor of T-test performance (β = −0.55, t = −4.23, *p* < 0.001). In contrast, fat mass was not a statistically significant predictor. For the U15 group, a multiple regression analysis was conducted to investigate whether height and fat mass predicted T-test performance. The overall regression model was statistically significant, F(2,45) = 4.26, *p* < 0.05, indicating that the predictors significantly explained variance in T-test performance. The model yielded an adjusted R^2^ = 0.122, showing that 12.2% of the variance in T-test performance was explained by the independent variables. Regarding the individual predictors, fat mass was a significant positive predictor of T-test performance (β = 0.40, t = 2.82, *p* < 0.01). In contrast, height was not a statistically significant predictor. This information is depicted in Table 8.

As far as Y-TEST CODS (right) is concerned in U13 a multiple regression analysis was conducted to examine whether FFM and fat mass predicted performance on the Y-TEST CODS (right side). The overall model was statistically significant, F(2,38) = 8.74, *p* < 0.001 indicating that the predictors significantly explained variance in change-of-direction performance. The model showed an adjusted R^2^ = 0.279, meaning that 27.9% of the variance in the Y-TEST CODS was explained by the predictors. Regarding the individual predictors, fat-free mass was a significant negative predictor of performance (β = −0.67, t = −4.18, *p* < 0.001). In contrast, fat was a significant positive predictor (β = 0.39, t = 2.42, *p* < 0.05). For U15 the overall model was statistically significant, F(2,45) = 6.55, *p* < 0.01. The model showed an adjusted R^2^ = 0.191, meaning that 19.1% of the variance in Y-TEST CODS (right) was explained by the independent variables. Regarding the individual predictors, fat was a significant positive predictor of performance (β = 0.74, t = 3.61, *p* < 0.001), whereas fat-free mass was a significant negative predictor (β = −0.54, t = −2.63, *p* < 0.05). For the U13 group, a multiple regression analysis was conducted to examine whether FFM and fat predicted performance on the Y-TEST CODS (left side). The overall model was statistically significant, F(2,38) = 5.19, *p* < 0.01, indicating that the predictors significantly explained variance in change-of-direction performance. The model showed an adjusted R^2^ = 0.173, meaning that 17.3% of the variance in the Y-TEST CODS (left) was explained by the independent variables. Regarding the individual predictors, FFM was a significant negative predictor of performance (β = −0.55, t = −3.22, *p* < 0.01). In contrast, fat was not a statistically significant predictor. For U15 the overall regression model was statistically significant, F(2,45) = 5.38, *p* < 0.01, indicating that the predictors significantly explained variance in change-of-direction performance. The model showed an adjusted R^2^ = 0.157, meaning that 15.7% of the variance in Y-TEST CODS (left) was explained by the independent variables. Regarding the individual predictors, fat mass was a significant positive predictor of performance (β = 0.68, t = 3.24, *p* < 0.01). In contrast, fat-free mass was a significant negative predictor (β = −0.59, t = −2.81, *p* < 0.01). For U13 group, a multiple regression analysis was conducted to examine whether height and fat mass predicted performance on the Y-TEST CODS BEST. The overall model was statistically significant, F(2,38) = 6.14, *p* < 0.01, indicating that the predictors significantly explained variance in change-of-direction performance. The model showed an R = 0.494, with an adjusted R^2^ = 0.204, meaning that 20.4% of the variance in Y-TEST CODS BEST was explained by the independent variables. Regarding the individual predictors, height was a significant negative predictor of performance (β = −0.53, t = −3.50, *p* < 0.001). In contrast, fat mass was not a statistically significant predictor. For the U15 group the overall regression model was not statistically significant, F(2,45) = 2.60, *p* = 0.085, indicating that the predictors did not significantly explain variance in change-of-direction performance. This information is depicted in Table 9.

## 4. Discussion

The present study sought to examine the predictive role of morphological characteristics on motor performance in youth basketball players, with particular focus on developmental differences between U13 and U15 age groups. The descriptive findings confirmed expected age-related patterns, as older athletes (U15) exhibited greater height, body mass, and fat-free mass, accompanied by superior performance in jumping, sprinting, and agility tasks. These observations are in line with the existing literature emphasizing the pivotal role of age on performance indices, whereby increases in height and lean mass enhance power-generating capacity [8,34]. Such physiological adaptations provide a plausible explanation for the performance advantages consistently observed in older athletes during development [35]. These findings may reflect not only chronological age differences but also maturational advances, including increases in androgen concentrations and muscle cross-sectional area [36]. Consequently, superior performance in the U15 group is likely the result of an interaction between biological maturation and accumulated training exposure rather than age alone.

The correlation analyses further revealed a distinct age-dependent differentiation in the relationships between morphological variables and motor performance. In the U13 cohort, height and fat-free mass were positively associated with jump and sprint ability, whereas body fat was consistently negatively associated with these performance indices. The stronger associations observed in U13 athletes suggest that physical performance during early adolescence remains highly dependent on structural and morphological characteristics, as the neuromuscular system is still developing and less influenced by accumulated training experience [37]. At this stage, technical proficiency and sport-specific adaptations may not yet be sufficiently developed to compensate for morphological disadvantages. Moreover, the detrimental impact of adiposity on performance outcomes is well established, with previous findings indicating that increased fat mass compromises both speed and change-of-direction speed, likely due to reduced relative power output and increased inertial demands [35]. In contrast, the weaker relationships observed in the U15 group may indicate a progressive shift toward a more complex performance model, where technical proficiency, neuromuscular efficiency, and training adaptations begin to outweigh the influence of basic morphological traits [37]. This transition likely coincides with puberty-related increases in muscle mass, tendon stiffness, and movement efficiency, all of which contribute to enhanced athletic performance independent of morphology alone [38].

The regression analyses provide further insight into this developmental transition. In the U13 group, morphological variables collectively accounted for a considerable proportion of variance in power performance outcomes, particularly in sprinting and jumping tasks, despite the absence of consistently significant individual predictors. This finding may indicate multicollinearity among body composition variables, confirmed via variance inflation factor in similar youth cohorts [37]. Beyond statistical considerations, however, these findings suggest that athletic performance in younger players is unlikely to be determined by a single morphological characteristic. Rather, performance appears to emerge from the combined interaction of body size, body composition, and development-related adaptations. This may explain why several predictors collectively explained a substantial proportion of variance, despite individual predictors not always reaching statistical significance. Notably, fat-free mass and body fat emerged as central contributors, highlighting the importance of body composition over generalized indices such as body mass. From a practical perspective, these findings suggest that coaches and practitioners should evaluate multiple morphological characteristics simultaneously rather than relying on isolated indicators when assessing athletic potential in younger basketball players. This interpretation is consistent with previous evidence demonstrating that lean mass is positively associated with muscular power production, whereas excess fat mass imposes both mechanical and metabolic limitations on movement efficiency [39,40].

In the U15 group, the regression models identified more discrete and specific predictors of performance, with body fat consistently emerging as a determinant with negative impact on both jumping and agility performance. These findings corroborate the literature indicating that elevated adiposity adversely affects performance and probably movement economy, particularly in high-intensity, weight-bearing activities [35]. This is also in line with recent biomechanical analyses, which further indicate that excess fat mass alters sprint kinematics and increases ground contact time, thereby impairing acceleration efficiency in young athletes [41]. Furthermore, greater body mass was associated with improved sprint performance, as indicated by lower sprint times, suggesting that increases in body mass, likely reflecting higher FFM, may enhance force production and acceleration capacity. However, body fat percentage and fat-free mass appear to provide more meaningful information regarding the relationship between morphology and performance in adolescent basketball players [39].

A similar developmental pattern was observed in CODS and RA performance. In the U13 group, morphological factors such as height, body mass, and fat-free mass were significantly associated with enhanced CODS ability, particularly under preplanned conditions. This finding suggests that greater structural characteristics facilitate more efficient execution of directional changes in younger athletes. Previous research supports the importance of such morphological determinants during early developmental stages, where physical attributes largely underpin performance capabilities [35,37]. Conversely, in the U15 group, body fat emerged as the primary predictor of diminished change-of-direction speed performance, underscoring the negative impact of excess fat mass during tasks requiring rapid deceleration and re-acceleration. The lack of significant predictors for RA measures in both age groups further suggests that these tasks are probably more strongly influenced by perceptual-cognitive processes, including decision-making and reaction to external stimuli, rather than purely physical characteristics [16,42]. This distinction between CODS and RA provides further support for contemporary theoretical models proposing that agility is a multifactorial construct in which perceptual-cognitive processes play a greater role than morphological attributes. Consequently, improving RA in youth basketball players may require training approaches that integrate stimulus perception, anticipation, and decision-making demands rather than focusing exclusively on physical development. Such findings also suggest that talent identification programs relying predominantly on morphological or physical performance measures may overlook athletes with superior perceptual-cognitive abilities.

Although our findings indicate that morphological characteristics were more strongly associated with CODS than with RA, previous studies have not always reported such clear distinctions. Some investigations have found moderate associations between morphological characteristics and RA performance, particularly in highly trained youth athletes, suggesting that physical qualities may indirectly contribute to reactive tasks through enhanced acceleration and braking capacities [33,43]. Differences in age, maturation status, competitive level, and agility-testing protocols may partly explain these inconsistencies [14]. In the present study, the absence of significant morphological predictors of RA supports the notion that, beyond a minimum physical threshold, successful performance in reactive tasks may depend increasingly on perceptual-cognitive processes, including stimulus recognition, anticipation, visual search strategies, and decision-making speed [14].

Collectively, these findings suggest that the contribution of morphological characteristics to athletic performance is not static across adolescence but evolves with development and training experience [17]. While performance in younger athletes appears to be strongly influenced by body size and body composition, older athletes demonstrate a more specialized profile in which body fat emerges as the primary morphological determinant, whereas other aspects of performance become increasingly influenced by neuromuscular, technical, and perceptual-cognitive factors [36]. The present study extends the previous research by simultaneously examining multiple morphological predictors across different performance domains and developmental stages, while also distinguishing between change-of-direction speed and RA. This approach provides a more comprehensive understanding of the mechanisms underlying youth basketball performance and offers practical guidance for talent identification and long-term athlete development.

Despite the valuable insights provided, several limitations of this study should be acknowledged. First, the cross-sectional design limits the ability to infer causal relationships between morphological characteristics and motor performance; longitudinal studies are recommended to better capture developmental trajectories. Second, the sample consisted exclusively of male basketball players, restricting generalizability to female athletes or other sports. Third, RA measures may have been influenced by cognitive and perceptual factors that were not directly assessed. Fourth, the present study focused on neuromuscular performance outcomes (jumping, sprint speed, and agility-related tasks) and did not assess physiological capacities relevant to basketball repeated high-intensity efforts and recovery, such as glycolytic and oxidative function, repeated sprint ability, intermittent endurance, or laboratory-based measures of anaerobic and aerobic power. Consequently, the physiological interpretation of the morphological predictors is limited to neuromuscular/explosive and directional performance and should not be generalized to metabolic performance or recovery capacity. Fifth, biological maturation was estimated using the maturity offset equation proposed by Moore et al. [27] from which age peak height velocity was derived. Although this method is widely used in youth sport research because it is non-invasive and practical for field settings, it provides only an indirect estimate of maturation status and is associated with potential error and bias, particularly in early- and late-maturing individuals. Therefore, the maturity indicator should be interpreted with caution and only as an approximate estimate of biological maturation. Furthermore, biological maturation was not a primary variable within the design of the present study and was not included in the main analyses. Given the well-established influence of maturation on morphological characteristics and physical performance during adolescence, its exclusion may limit the explanatory power of the models. However, maturation was not included in the original study design, which focused on readily available morphological characteristics for practical application in youth sport settings. We did not use a maturity-based grouping (e.g., estimated age at peak height velocity) because the primary aim was to compare established competitive age categories (U13 vs. U15), which are commonly used in practice and selection. However, future studies should include maturity indicators to better separate maturity-related from age-related group effects. Finally, another limitation of the present study is the relatively small sample size within each age group in relation to the number of predictors included in the regression analyses. Therefore, the regression models should be interpreted as exploratory, and the findings should be considered preliminary rather than definitive.

From an applied perspective, the findings of the present study highlight the importance of morphological characteristics, particularly body composition, in influencing motor performance in youth basketball players, with distinct age-related differences. Therefore, in the U13 group, practitioners should prioritize training strategies that support the development of lean body mass while controlling excess adiposity, as these factors appear to underpin performance across multiple physical domains. In the U15 group, where fewer morphological variables were associated with performance, practitioners should place particular emphasis on maintaining optimal body composition, especially by minimizing excess body fat, to avoid impairments in explosive and agility-related performance.

## 5. Conclusions

The findings of the present study indicate a distinct developmental shift in the determinants of motor performance among young male basketball players. During early adolescence (U13), performance appears to be primarily influenced by morphological characteristics, particularly body composition and structural attributes such as height and fat-free mass. At this stage, physical development and morphological advantages play a dominant role in shaping athletic capabilities [8]. However, as athletes transition into mid-adolescence (U15), the influence of these factors becomes less pronounced, giving way to a more complex and multifactorial performance model.

In this later stage, maturity, training experience, and cognitive-perceptual skills probably emerge as increasingly critical contributors to performance [42], but this assumption remains to be confirmed. This progression during mid-adolescence reflects the natural course of athletic development, where growth and development provide the initial foundation. However, performance differentiation seems to be ultimately driven by the integration of physical, technical, and cognitive components.

## Figures and Tables

**Table 1:** Descriptive statistics for age, maturity, and training age.

Variables	Age Group	Mean ± SD	Skewness	Kurtosis	Minimum	Maximum
Age (years)	U13	11.79 ± 0.79	0.08	−1.39	10.44	12.97
	U15	14.17 ± 0.54	−0.07	−1.21	13.17	14.99
	Total	13.07 ± 1.36	−0.33	−1.19	10.44	14.99
Maturity (years from PHV)	U13	−1.24 ± 0.79	0.37	−1.13	−2.57	0.24
	U15	0.83 ± 0.61	−0.13	−0.20	−0.59	2.14
	Total	−0.12 ± 1.25	−0.26	−1.13	−2.57	2.13
Training Age (years)	U13	4.96 ± 2.33	−0.35	−1.05	0.50	8
	U15	6.34 ± 1.97	−0.75	−0.19	1	10
	Total	5.71 ± 2.24	−0.59	−0.51	0.50	10

Note: PHV = Peak Height Velocity.

**Table 2:** Descriptive statistics for morphological characteristics.

Variables	Age Group	Mean ± SD	Skewness	Kurtosis	Minimum	Maximum
Body Mass (kg)	U13	49.91 ± 10.2	0.53	0.03	34.40	74.80
	U15	62.83 ± 12.65	0.61	0.75	36.10	96.60
	Total	56.88 ± 13.21	0.61	0.52	34.40	96.60
Height (cm)	U13	158.46 ± 9.87	0.38	−1.12	144.0	176.0
	U15	172.46 ± 7.63	−0.32	0.37	153.0	189.0
	Total	166.01 ± 11.16	−0.27	−0.88	1.44	1.89
BMI (kg/m^2^)	U13	19.71 ± 2.53	0.66	0.19	15.49	26.35
	U15	20.98 ± 3.16	0.56	−0.27	15.42	28.37
	Total	20.39 ± 2.94	0.68	0.05	15.40	28.40
Fat (kg)	U13	20.00 ± 4.65	0.20	−1.06	12.77	28.96
	U15	18.90 ± 5.11	0.48	−0.57	10.82	29.65
	Total	19.41 ± 4.91	0.33	−0.82	10.82	29.65
FFM (%)	U13	39.84 ± 7.76	0.56	−0.31	28.40	59.40
	U15	50.47 ± 7.72	0.08	0.89	31.50	71.48
	Total	45.57 ± 9.36	0.13	−0.38	28.40	71.48

Note: FFM = Fat Free Mass.

**Table 3:** Descriptive statistics for Jumps, Sprints, and T-test.

Variables	Age Group	Mean ± SD	Skewness	Kurtosis	Minimum	Maximum
SJ (cm)	U13	26.39 ± 5.20	−0.19	−1.05	16.40	34.62
	U15	28.46 ± 5.42	0.28	−0.06	18.40	43
	Total	27.51 ± 5.39	0.09	−0.29	16.40	43
CMJ (cm)	U13	29.57 ± 6.18	−0.18	−1.33	17.70	40
	U15	31.18 ± 5.35	0.32	−0.17	20.84	45.1
	Total	30.44 ± 5.77	−0.02	−0.67	17.70	45.10
Sprint 10m (s)	U13	2.17 ± 0.17	0.68	0.44	1.89	2.60
	U15	1.96 ± 0.12	0.08	−0.28	1.75	2.25
	Total	2.06 ± 0.18	0.74	0.83	1.75	2.60
Sprint 20m (s)	U13	3.80 ± 0.27	0.63	0.09	3.35	4.46
	U15	3.46 ± 0.20	0.11	−0.34	3.11	3.97
	Total	3.62 ± 0.29	0.59	0.42	3.11	4.46
T-Test CODS (s)	U13	12.25 ± 1.05	0.14	−0.54	10.49	14.89
	U15	10.76 ± 0.65	0.61	0.87	9.53	12.78
	Total	11.44 ± 1.13	0.68	−0.17	9.53	14.89

Note: SJ = Squat Jump, CMJ = Countermovement Jump.

**Table 4:** Descriptive statistics for the Preplanned and Random protocol in the Y-test and Stop ‘n go test.

Variables	Age Group	Mean ± SD	Skewness	Kurtosis	Minimum	Maximum
YTESTCODSRIGHT (s)	U13	2.24 ± 0.19	1.41	4.49	1.94	2.98
	U15	2.04 ± 0.12	1.56	6.23	1.81	2.53
	Total	2.13 ± 0.18	1.47	4.11	1.81	2.98
YTESTCODSLEFT (s)	U13	2.27 ± 0.18	0.03	−0.51	1.95	2.67
	U15	2.03 ± 0.12	0.85	2.08	1.81	2.43
	Total	2.14 ± 0.19	0.64	−0.27	1.81	2.67
YTESTCODSBEST (s)	U13	2.20 ± 0.15	0.15	−0.58	1.94	2.54
	U15	2.00 ± 0.11	1.15	4.14	1.81	2.43
	Total	2.09 ± 0.16	0.67	−0.09	1.81	2.54
YTESTRARIGHT (s)	U13	3.28 ± 0.33	0.29	0.97	2.49	4.16
	U15	2.89 ± 0.26	0.36	−0.11	2.34	3.54
	Total	3.07 ± 0.35	0.48	0.33	2.34	4.16
YTESTRALEFT (s)	U13	3.26 ± 0.31	0.45	−0.34	2.73	3.96
	U15	2.83 ± 0.29	−0.48	−0.01	2.13	3.29
	Total	3.03 ± 0.37	0.13	0.29	2.13	3.96
YTESTRABEST (s)	U13	3.14 ± 0.26	−0.08	0.50	2.49	3.76
	U15	2.77 ± 0.27	−0.31	0.05	2.13	3.29
	Total	2.94 ± 0.32	−0.14	0.03	2.13	3.76
REAC-INDEXYTEST	U13	0.94 ± 0.24	−0.02	−0.62	0.40	1.41
	U15	0.77 ± 0.24	−0.16	0.25	0.21	1.28
	Total	0.85 ± 0.25	−0.08	−0.09	0.21	1.41
STOPGOBEST CODS (s)	U13	7.94 ± 0.61	0.29	−0.02	6.50	9.33
	U15	7.11 ± 0.46	1.29	2.10	6.37	8.68
	Total	7.49 ± 0.67	0.67	−0.16	6.37	9.33
STOPGOBESTRA (s)	U13	10.25 ± 0.72	0.09	−0.61	8.79	11.79
	U15	8.75 ± 0.64	0.62	−0.25	7.65	10.35
	Total	9.44 ± 1.01	0.28	−0.84	7.65	11.79
REAC-INDEXSTOPGO	U13	2.31 ± 0.58	0.32	−0.73	1.42	3.60
	U15	1.65 ± 0.56	0.42	0.04	0.48	3.13
	Total	1.95 ± 0.66	0.29	−0.36	0.48	3.60

Note: YTESTCODSRIGHT: Y-test planned execution to the right, YTESTCODSLEFT: Y-test planned execution to the left, YTESTCODSBEST: Y-test best planned execution, YTESTRARIGHT: Y-test random execution to the right, YTESTRALEFT: Y-test random execution to the left, YTESTRABEST: Y-test best random execution. REAC-INDEXYTEST (reactive index): YTESTRABEST-YTESTCODSBEST, STOPGOBESTCODS: Stop ‘n go test planned protocol, STOPGOBESTRA: Stop ‘n go test random protocol, REACINDEXSTOPGO (reactive index): STOPGOBESTRA-STOPGOBESTCODS.

**Table 5:** Correlations among morphological characteristics with Jump, Sprint, and T-test CODS in U13 and U15 group.

Variables	Age Groups	SJ	CMJ	Sprint 10 m	Sprint 20 m	T-Test CODS
Body Mass	U13	0.20 ns	0.16 ns	−0.41 **	−0.33 *	−0.45 **
	U15	−0.09 ns	−0.25 ns	−0.07 ns	−0.07 ns	0.29 *
Height	U13	0.39 *	0.39 *	−0.47 **	−48 ***	−0.57 ***
	U15	0.07 ns	0.03 ns	−0.14 ns	−0.20 ns	0.10 ns
Fat	U13	−0.47 **	−0.49 ***	0.35 *	0.46 **	0.25 ns
	U15	−0.36 *	−0.48 **	0.18 ns	0.25 ns	0.40 **
FFM	U13	0.36 *	0.32 *	−0.53 ***	−0.48 ***	−0.55 ***
	U15	0.05 ns	−0.10 ns	−0.19 ns	−0.22 ns	0.18 ns

Note. SJ = Squat Jump, CMJ = Countermovement Jump, FFM = Fat Free Mass, * *p* < 0.05, ** *p* < 0.01, *** *p* < 0.001, ns = non-significant.

**Table 6:** Correlations among morphological characteristics with Time for preplanned and random agility protocol in U13 and U15 group.

Variables	Age Group	Y-TEST CODS RIGHT	Y-TEST CODS LEFT	Y-TEST CODS BEST	Y-TEST RA RIGHT	Y-TEST RA LEFT	Y-TEST RA BEST	REAC-INDEX Y-TEST	STOP GO BEST CODS	STOP GO BEST RA	REAC-INDEX STOP GO
Body Mass	U13	−0.34 *	−0.31*	−0.37 *	−0.22 ns	−0.40 **	−0.36 *	−0.15 ns	−0.22 ns	−0.19 ns	−0.01 ns
	U15	0.17 ns	0.06 ns	0.04 ns	0.11 ns	0.11 ns	0.12 ns	0.12 ns	0.25 ns	0.14 ns	−0.04 ns
Height	U13	−0.46 **	−0.36 *	−0.47 **	−0.19 ns	−0.37 *	−0.37 *	−0.09 ns	−0.28 ns	−0.15 ns	0.12 ns
	U15	−0.00 ns	−0.10 ns	−0.11 ns	0.02 ns	0.03 ns	0.01 ns	0.07 ns	0.10 ns	0.17 ns	0.11 ns
Fat	U13	0.36 *	0.26 ns	0.30 ns	0.20 ns	0.07 ns	0.20 ns	0.02 ns	0.18 ns	−0.02 ns	−0.20 ns
	U15	0.39 **	0.29 *	0.29 *	0.12 ns	0.19 ns	0.19 ns	0.09 ns	0.34 *	0.14 ns	−0.11 ns
FFM	U13	−0.46 **	−0.40 *	−0.47 **	−0.28 ns	−0.45 **	−0.44 **	−0.18 ns	−0.28 ns	−0.19 ns	0.05 ns
	U15	0.03 ns	−0.07 ns	−0.10 ns	0.06 ns	0.04 ns	0.06 ns	0.11 ns	0.16 ns	0.11 ns	−0.01 ns

Note (a). * *p* < 0.05, ** *p* < 0.01, ns = non-significant. Note (b). YTESTCODSRIGHT: Y-test planned execution to the right, YTESTCODSLEFT: Y-test planned execution to the left, YTESTCODSBEST: Y-test best planned execution, YTESTRARIGHT: Y-test random execution to the right, YTESTRALEFT: Y-test random execution to the left, YTESTRABEST: Y-test best random execution. REAC-INDEXYTEST (reactive index): YTESTRABEST-YTESTCODSBEST, STOPGOBESTCODS: Stop ‘n go test planned protocol, STOPGOBESTRA: Stop ‘n go test random protocol, REACINDEXSTOPGO (reactive index): STOPGOBESTRA-STOPGOBESTCODS. Note (c). In time-based tests, lower scores indicate superior performance; therefore, negative correlations reflect better performance outcomes.

**Table 7:** Multiple regression analyses for predicting SJ and CMJ performance in U13 and U15 athletes.

DV	Age Group	Predictor	B	SE	β	t	*p*	95% CI for B	R^2^	Adjusted R^2^	F
SJ	U13	Body Mass	−0.38	1.25	−0.75	−0.31	0.762	[−2.92, 2.16]	0.361	0.290	5.09 **
		Height	0.01	0.16	0.02	0.06	0.951	[−0.31, 0.33]			
		FAT	−0.29	0.81	−0.26	−0.36	0.724	[−1.93, 1.35]			
		FFM	0.71	1.61	1.07	0.44	0.660	[−2.55, 3.98]			
	U15	Body Mass	−0.84	0.78	−1.95	−1.07	0.292	[−2.42, 0.75]	0.279	0.212	4.16 **
		Height	−0.29	0.19	−0.41	−1.57	0.124	[−0.66, 0.08]			
		Fat	−0.04	0.68	−0.04	−0.06	0.951	[−1.41, 1.33]			
		FFM	1.60	1.02	2.28	1.57	0.123	[−0.45, 3.65]			
CMJ	U13	Height	0.21	0.08	0.33	2.53	0.016	[0.04, 0.38]	0.350	0.315	10.21 ***
		Fat	−0.60	0.18	−0.45	−3.43	0.001	[−0.96, −0.25]			
	U15	Height	0.11	0.09	0.15	1.14	0.259	[−0.08, 0.29]	0.251	0.218	7.55 ***
		Fat	−0.50	0.14	−0.48	−3.70	0.001	[−0.77, −0.23]			

Note. ** *p* < 0.01, *** *p* < 0.001.

**Table 8:** Multiple regression analyses for predicting Sprint 10, Sprint 20 and T-test (CODS) performance in U13 and U15 athletes.

DV	Age Group	Predictor	B	SE	β	t	*p*	95% CI for B	R^2^	Adjusted R^2^	F
Sprint 10	U13	Body Mass	−0.07	0.04	−3.91	−1.75	0.088	[−0.14, 0.10]	0.471	0.412	8.00 ***
		Height	0.00	0.01	0.09	0.34	0.737	[−0.01, 0.01]			
		Fat	0.06	0.02	1.52	2.32	0.026	[0.01, 0.10]			
		FFM	0.07	0.05	3.09	1.41	0.167	[−0.03, 0.16]			
	U15	Body Mass	0.03	0.02	3.50	1.89	0.065	[−0.00, 0.07]	0.264	0.195	3.85 **
		Height	0.01	0.00	0.47	1.78	0.082	[−0.00, 0.02]			
		Fat	−0.01	0.02	−0.61	−0.95	0.350	[−0.04, 0.02]			
		FFM	−0.05	0.02	−3.57	−2.44	0.019	[−0.10, −0.01]			
Sprint 20	U13	Body mass	−0.01	0.00	−0.53	−4.24	0.000	[−0.02, 0.01]	0.467	0.438	16.62 ***
		Fat	0.04	0.01	0.63	5.05	0.000	[0.02, 0.05]			
	U15	Body mass	−0.01	0.00	−0.66	−3.20	0.003	[−0.02, −0.00]	0.237	0.203	7.00 **
		Fat	0.03	0.01	0.76	3.70	0.001	[0.01, 0.05]			
T-test	U13	Height	−0.06	0.01	−0.55	−4.23	0.000	[−0.09, −0.02]	0.363	0.330	10.83 ***
		Fat	0.04	0.03	0.19	1.44	0.157	[−0.03, 0.10]			
	U15	Height	0.00	0.01	0.00	0.02	0.98	[−0.02, 0.02]	0.159	0.122	4.26 **
		Fat	0.05	0.02	0.40	2.82	0.01	[0.01, 0.09]			

Note. ** *p* < 0.01, *** *p* < 0.001.

**Table 9:** Multiple regression analyses for predicting Y-TEST right, left, and best performance in U13 and U15 athletes.

DV	Age Group	Predictor	B	SE	β	t	*p*	95% CI for B	R^2^	Adjusted R^2^	F
Y-TEST CODS RIGHT	U13	Fat	0.02	0.01	0.39	2.42	0.020	[0.00, 0.04]	0.315	0.279	8.74 ***
		FFM	−0.02	0.00	−0.67	−4.18	0.000	[−0.02, −0.01]			
	U15	Fat	0.02	0.00	0.74	3.61	0.001	[0.01, 0.02]	0.226	0.191	6.55 **
		FFM	−0.01	0.00	−0.54	−2.63	0.012	[−0.02, −0.00]			
Y-TEST CODS LEFT	U13	Fat	0.01	0.01	0.29	1.69	0.100	[−0.00, 0.03]	0.215	0.173	5.19 **
		FFM	−0.01	0.00	−0.55	−3.22	0.003	[−0.02, −0.01]			
	U15	Fat	0.01	0.00	0.68	3.24	0.002	[0.01, 0.02]	0.193	0.157	5.38 **
		FFM	−0.01	0.00	−0.59	−2.81	0.007	[−0.02, −0.00]			
Y-TEST CODS BEST	U13	Height	−0.01	0.00	−0.53	−3.50	0.001	[−0.01, −0.00]	0.244	0.204	6.14 **
	U15	Fat	0.01	0.01	0.17	1.09	0.281	[−0.01, 0.02]			
	U13	Height	−0.00	0.00	−0.27	−1.70	0.096	[−0.01, 0.00]	0.104	0.064	2.60
	U15	Fat	0.01	0.00	0.34	2.13	0.039	[0.00, 0.01]			

Note. ** *p* < 0.01, *** *p* < 0.001.

## Data Availability

The data that supports the results of this study are available from the corresponding author upon reasonable request, as they cannot be shared publicly due to ethical restrictions.

## Abbreviations

The following abbreviations are used in this manuscript:

CODS	Change of Direction Speed
FFM	Fat-free mass
RA	Reactive agility
SJ	Squat Jump
CMJ	Countermovement Jump
